# Intercentre reproducibility of second eigenvector orientation in cardiac diffusion tensor imaging

**DOI:** 10.1186/1532-429X-18-S1-P35

**Published:** 2016-01-27

**Authors:** Elizabeth M Tunnicliffe, Pedro Ferreira, Andrew D Scott, Rina Ariga, Laura-Ann McGill, Sonia Nielles-Vallespin, Stefan Neubauer, Dudley J Pennell, Matthew D Robson, David Firmin

**Affiliations:** 1 grid.4991.50000000419368948OCMR, University of Oxford, Oxford, UK; 2 grid.439338.6NIHR Cardiovascular BRU, Royal Brompton Hospital & Imperial College, London, UK; 3 grid.279885.90000000122934638National Institutes of Health, National Heart Lung and Blood Institute, Bethesda, MD USA

## Background

Diffusion tensor imaging enables the study of cardiac microstructure in vivo, including its changes through the cardiac cycle. The left ventricle consists of myocytes with opposing helical arrangements in the epi- and endocardium. Histology shows that these myocytes are arranged into small laminar "sheetlet" structures, separated by shear layers which allow the sheetlets to move and reorient relative to each other during myocardial contraction. While the direction of the first eigenvector of the cardiac diffusion tensor indicates the average direction of the myocytes in a voxel, the second eigenvector appears to indicate the mean orientation of the sheetlets (1). Recent work has demonstrated that there are differences between the motion of these sheetlets between healthy volunteers and patients with hypertrophic cardiomyopathy (2). In order to facilitate comparison of sheetlet orientations in disease between different centres, the aim of this study was to test the intercentre reproducibility of the second eigenvector orientation.

## Methods

Ten healthy volunteers were scanned at two centres using a stimulated echo cardiac diffusion tensor sequence (3) on different models of 3T MRI scanner. The two centres calculated the second eigenvector angle (E2A) for each volunteer in both systole and diastole in a single mid-ventricular slice using scripts written independently in Matlab (Mathworks, Natick, MA). The mean magnitude of E2A (denoted |E2A|) was calculated over similarly drawn (3) ROIs covering the left ventricle to produce a single number for each subject in systole and diastole. Bland-Altman analysis was carried out on the values for each centre, and statistical significance of differences tested using a paired t-test. The mean intrasubject, intercentre coefficient of variance was calculated.

## Results

**Figure 1:**
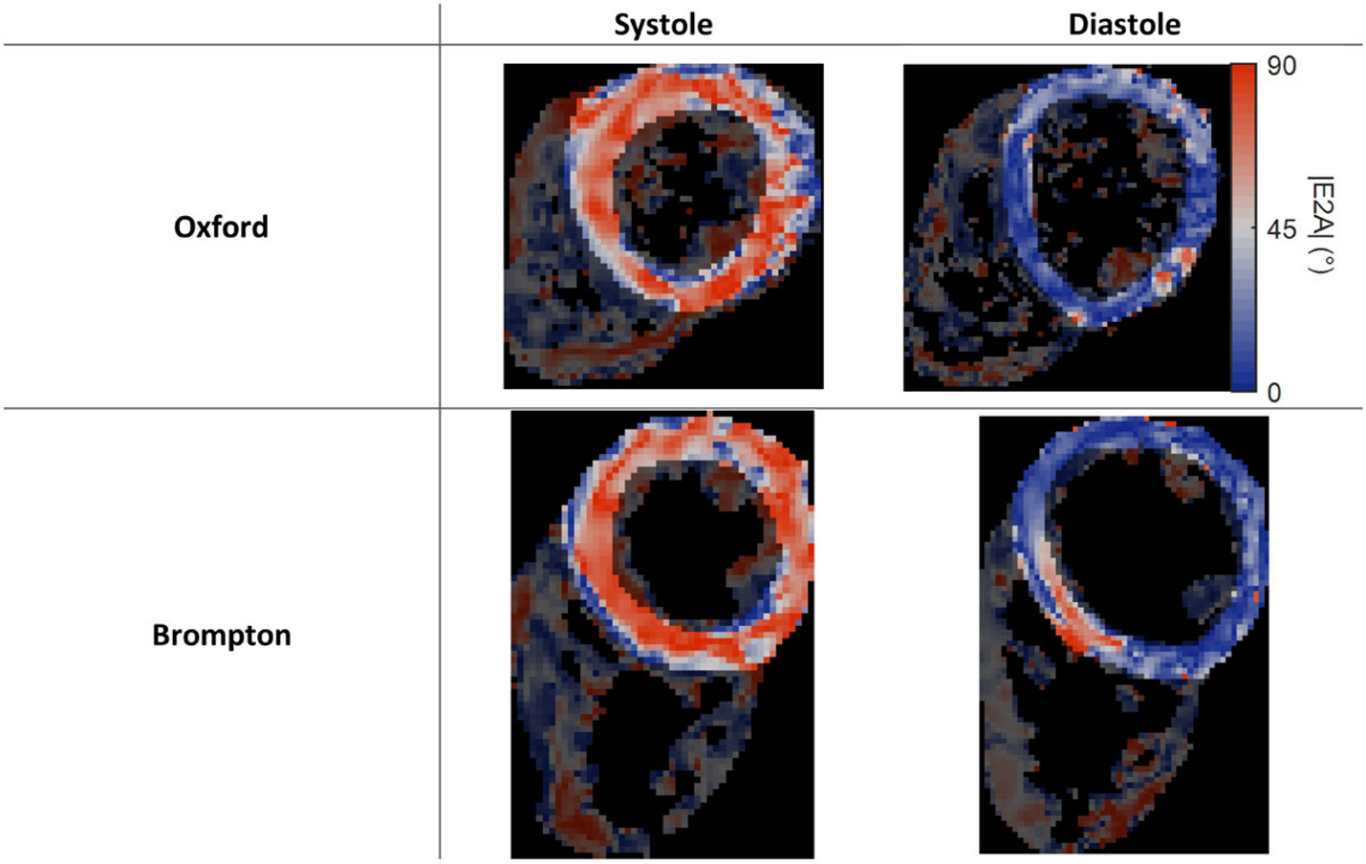
**Example |E2A| maps from both centres in systole and diastole for one of the normal volunteers**.

**Figure 2:**
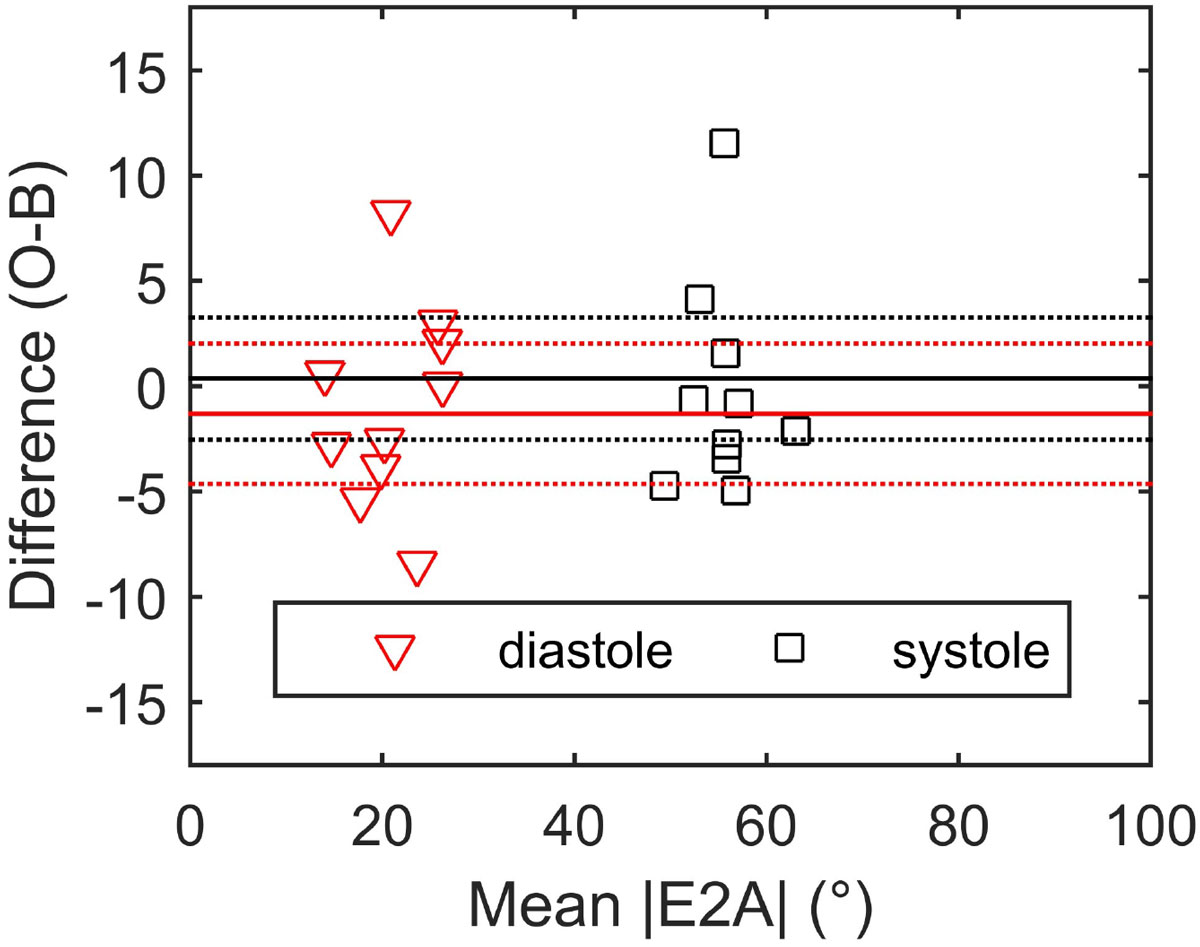
**A Bland-Altman plot showing the differences between the |E2A| measured at the two centres**.

Example maps for one of the volunteers are shown in Figure 1. Mean |E2A| ± standard deviation for all subjects averaged over both centres was 55 ± 4° in systole and 21 ± 5° in diastole. A Bland-Altman plot comparing the |E2A| measurements from the two centres is shown in Figure 2. The mean intercentre difference in systole was 0.2 ± 5° (p = 0.9) and in diastole was 0.9 ± 5° (p = 0.6), giving limits of agreement between 46° and 65° in systole and 12° and 30° in diastole. The intrasubject coefficient of variation was 7% for systolic |E2A| and 15% for diastolic |E2A|. The CoV is higher in diastole primarily because of the lower numeric value of the sheetlet angle in diastole, not because of higher variance in the measurements.

## Conclusions

In healthy volunteers, it is possible to obtain consistent evaluations of the second eigenvector angle, a measure of the myocardial sheetlet orientation, at two different centres running equivalent stimulated echo cardiac diffusion tensor sequences.
